# The history and current epidemiology of malaria in Kalimantan, Indonesia

**DOI:** 10.1186/s12936-022-04366-5

**Published:** 2022-11-14

**Authors:** Sri Riyati Sugiarto, J. Kevin Baird, Balbir Singh, Iqbal Elyazar, Timothy M. E. Davis

**Affiliations:** 1 University of Western Australia, Medical School, Fremantle Hospital, PO Box 480, Fremantle, WA Australia; 2 Eijkman Oxford Clinical Research Unit (EOCRU), Jalan Diponegoro 69, Jakarta, 10430 Indonesia; 3 Centre for Tropical Medicine and Global Health, Nuffield Department of Medicine, University of Oxford, Oxford, UK; 4 Malaria Research Centre, Universiti Malaysia Sarawak, 94300 Kota Samarahan, Sarawak Malaysia

**Keywords:** Kalimantan, Malaria, History, Epidemiology, *Plasmodium knowlesi*

## Abstract

Kalimantan is a part of Indonesia, which occupies the southern three-quarters of the island of Borneo, sharing a border with the Malaysian states of Sabah and Sarawak. Although most areas of Kalimantan have low and stable transmission of *Plasmodium falciparum* and *Plasmodium vivax*, there are relatively high case numbers in the province of East Kalimantan. Two aspects of malaria endemicity in Kalimantan differentiate it from the rest of Indonesia, namely recent deforestation and potential exposure to the zoonotic malaria caused by *Plasmodium knowlesi* that occurs in relatively large numbers in adjacent Malaysian Borneo. In the present review, the history of malaria and its current epidemiology in Kalimantan are examined, including control and eradication efforts over the past two centuries, mosquito vector prevalence, anti-malarial use and parasite resistance, and the available data from case reports of knowlesi malaria and the presence of conditions which would support transmission of this zoonotic infection.

**Supplementary Information:**

The online version contains supplementary material available at 10.1186/s12936-022-04366-5.

## Background

Indonesia, the fourth most populated country and occupying most of the largest archipelago in the world, had an estimated 800,000 malaria cases in 2021 according to the latest World Health Organization (WHO) report, the second highest number in South-east Asia after India [1]. Although the Indonesian government has targeted malaria elimination by 2030 [2], case numbers have remained relatively stable over the past 5 years [3]. Approximately 130 million Indonesians live in high risk regions [3–5], but the geographical distribution of transmission is highly heterogeneous [4]. Of the 514 districts and municipalities of Indonesia, 351 (68.3%) were certified free of malaria in 2022 [6]. In the remainder, and based on the most complete estimates, the prevalence varies from 0.02 to 12.07% [7]. Even though the majority of provinces have hypoendemic to mesoendemic malaria [3, 8], there is relatively intense transmission in eastern Indonesia [7, 9] including parts of Indonesian Borneo (Kalimantan) [7, 10].

**Fig. 1:**
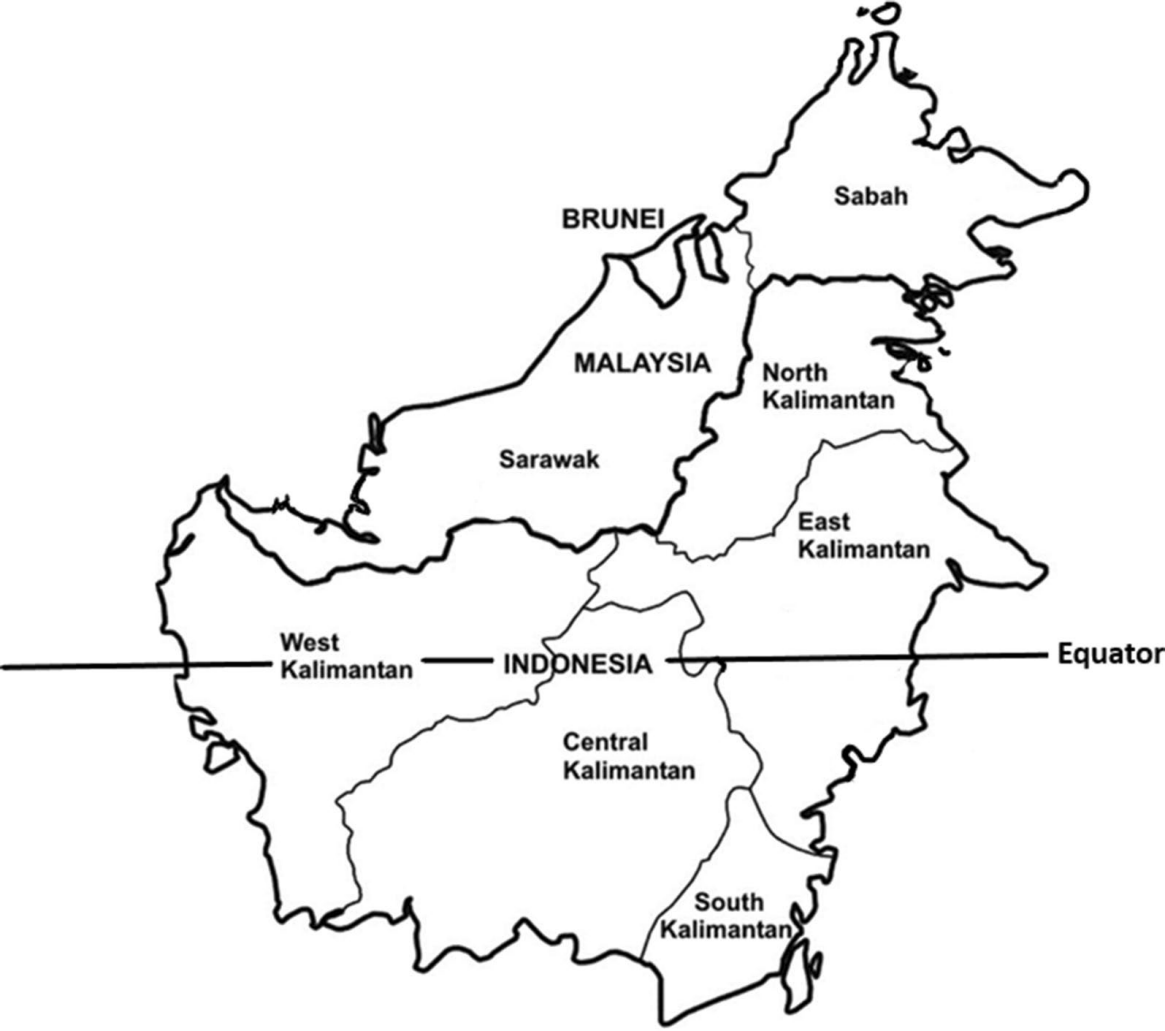
Kalimantan map showing its five provinces and relationship to Malaysian Borneo

Kalimantan occupies the southern three-quarters of the island of Borneo (Fig. 1). In the north, it shares a border with the Malaysian Borneo states of Sabah and Sarawak. Most areas of Kalimantan have low and stable transmission of the dominant *Plasmodium* species, *Plasmodium falciparum* and *Plasmodium vivax* [11]. The average Annual Parasite Index (API; number of positive cases per 1000 individuals in a year) is below 0.15 except for relatively high transmission areas in East Kalimantan [2, 11]. There are two aspects of malaria endemicity in Kalimantan that differentiate it from the rest of Indonesia. First, although the island of Borneo has one of the largest remaining forested areas in South-east Asia, about a third of Borneo has been deforested in the last 50 years [12] which, with increasing urbanization and climate change [13], has the potential to impact malaria transmission. Second, the zoonotic malaria caused by *Plasmodium knowlesi* is widespread in South-east Asia [14] and there are relatively large numbers of cases in both Sarawak [15–18] and Sabah [19–21]. Although *P. knowlesi* is the predominant cause of human malaria in Malaysian Borneo [22, 23], very few cases have been reported from Kalimantan despite its geographical proximity and shared ecologies.

Given this background, the aim of the present narrative review was to detail the recent history of malaria in Kalimantan including cases of *P. knowlesi*, and to examine geo-epidemiological trends that are likely to influence future malaria transmission in this large Indonesian territory.

## Methods

The present systematic review was conducted according to PRISMA 2020 guidelines [24]. A publication search was performed in PubMed (MEDLINE) and the Indonesian repository site www.neliti.com using the keywords “malaria + Kalimantan + Indonesia” and “plasmodium + Kalimantan”. The references cited in all eligible publications were reviewed to identify relevant articles that had been missed in the initial search. Supplementary malaria data were obtained from reports of Provincial Health Department compiled from monthly reports of Health Centres at village to sub-district and from sub-district to district level. Information on *Anopheles* vectors was collected from identified publications, and government and Global Fund reports. Information regarding malaria control measures in Kalimantan was collected from the provincial Health Department and personal communication with malaria officers.

### Geographical situation and population

Kalimantan is located at 1S 114°E, has a total area of 539,238 square km [25], and is crossed by the equator [25] (Fig. 1). It is connected to Malaysian Borneo by roads from West, East and North Kalimantan. About 77% of Kalimantan is state forest land, including a million hectares of peat land where a large portion of primary rainforest has been converted to palm-oil plantations and timber logging areas [26]. The total population in 2021 was 16.8 million (representing approximately 6% of the total Indonesian population) with most inhabitants aged between 15 and 50 years [27]. The average annual population growth rate was 2.12% in 2020 [27].

Kalimantan consists of five provinces (*propinsi*) (Fig. 1): Central Kalimantan (*Kalimantan Tengah,* capital city Palangkaraya, area 154,000 km^2^, population 2.65 million), East Kalimantan (*Kalimantan Timur,* capital city Samarinda, area 127,300 km^2^, population 3.72 million), North Kalimantan (*Kalimantan Utara,* capital city Tanjung Selor, area 154,000 km^2^, population 0.072 million), South Kalimantan (*Kalimantan Selatan,* capital city Banjarbaru, area 38,700 km^2^, population 4.31 million), and West Kalimantan (*Kalimantan Barat,* capital city Pontianak, area 147.300 km^2^, population 5.07 million) [25, 27, 28]. Most of the population engages in agricultural and forest-related industries, but other economic activities include mining, fisheries, government agencies and trade [27].

### History of malaria in Kalimantan

#### Early reports of malaria

Dutch traders arrived in Indonesia in the late 16^th^ Century and the Dutch colonized the archipelago over the next two centuries. Early letters and reports from Dutch traders indicated that a certain type fever, very likely malaria, was a significant health problem affecting military activity and trade [29]. As the Indonesian central government and the administration of the Dutch East Indies Company (Vereenigde Oost-Indische Company; VOC) were located on the island of Java, most early Indonesian malaria-related publications were from Java. These included a cross-sectional comparison of malaria prevalence and parasite density in two major cities [30] and a subsequent study in the 18th Century in Batavia (now Jakarta, the country’s capital) that showed high morbidity from tertian or continual fevers that claimed thousands of VOC employees’ lives each year [31]. In a report to the German Colonial Office in 1899, Robert Koch investigated aspects of malaria in adults, (including military hospitals) and children in Java [30, 32]. Subsequently, the Netherland East Indies ‘ethical policy’ (*ethische politiek*) that was meant to improve the welfare of indigenous Indonesians through irrigation, transmigration and education inadvertently ignited malaria outbreaks in Java in the first 30 years of the 20th Century by increasing transmission [33, 34].

Indonesian Borneo has been occupied by Dayak tribes since 242 BC and was colonized by Hindu, Buddhist and Muslim kingdoms over the following centuries before the arrival of European settlers [35]. Although it is likely that malaria was present in Kalimantan since the beginning of human settlement, documentation is sparse. Chinese merchants trading in South Kalimantan in 1660 declined the Sultan of Banjarmasin’s demand to work in the non-tidal zone of Martapura rather than in their usual location at Old Banjar due the possibility of contracting fatal ‘hot fevers’ [36, 37]. In 1786, many Dutch soldiers developed ‘febris intermittens’ during the first military intervention inland from Banjarmasin, and this malady was encountered repeatedly in subsequent military missions [37]. Dutch military health service reports from Kalimantan in 1860 indicated that approximately 20% of the patients in coastal city hospitals were suspected to suffer from malaria compared to almost 50% in inland hospitals [36, 37], highlighting the lower case load in tidal coastal zones compared to forested areas. However, during early European settlement, Kalimantan was considered as a relatively healthy place according to the first European explorer Schwaner in 1853 [38], presumably because intense malaria transmission was limited in the small, dispersed agricultural communities [36].

The first formal report of malaria in Kalimantan in the published medical literature was written by the Dutch physician Nieuwenhuis [36, 39]. While working in Sambas, West Kalimantan, between 1894 and 1898, he argued that malaria was more prevalent at higher altitudes and in sandy areas. He found only 6 malaria cases out of 2,103 children living on the alluvial plain compared to almost all (403 out of 420) children from the hilly inland area. This difference was suggested subsequently to be due to fluctuating tides and salinity adversely affected breeding of *Anopheles sundaicus* and *Anopheles beazai* mosquito vectors in low lying areas [40]. There were exceptions to Nieuwenhuis’ observations, however, in that the Apo-Kayan villages in hilly areas had a low incidence of malaria [41] while the nomadic tribes remained virtually malaria free [42]. For this latter group, their mobility was thought to inhibit the development of foci of local transmission [42]. In the case of the Apo-Kayan, it was suggested that habitation at high altitude and at a relatively low temperature, as well as the cultivation of dry rice, did not favour mosquito breeding [39, 43]. Nevertheless, population movement from coastal areas to the interior resulted in an increased incidence of malaria in the early 19th Century, a situation that is still relevant for malaria epidemiology in Kalimantan two centuries later.

#### Prevention and treatment timelines

The first drug used widely for both malaria treatment and prophylaxis was quinine, a short-acting alkaloid extracted from the bark of the cinchona tree that is native to South America [44, 45]. It was introduced in the 19th Century and Dutch cinchona plantations in Java became the most successful globally [46]. Indeed, the Dutch had a near monopoly on the supply of quinine and the *Bandoengsche Kininefabriek* company was the biggest quinine producer in the world in 1924 [47]. The drug had a significant role during the Second World War as the loss of soldiers due to malaria was greater than war-related injuries [48]. Worldwide quinine production diminished substantially after the Japanese Imperial Army invaded and occupied Indonesia in 1942. Chloroquine was synthesized in Germany in the 1930s as resochin and a wartime American effort saw it registered in 1946 as a less expensive, safer, and better tolerated therapy of acute malaria. Plantation-dependent quinine subsequently declined significantly because of a lack of demand [49].

In the mid-19th Century, a restricted amount of quinine was brought by the Dutch to coastal towns in Kalimantan at a time when native Bornean people employed various non-pharmacological ways of preventing and remedying the disease [36, 50]. The indigenous Dayaks built their longhouses on stilts above the maximum vertical flight range of mosquitoes, separated their villages by distances exceeding the horizontal flight range, and produced smoke around the house that deterred mosquito bites [36, 51, 52]. Domesticated animals such as pigs, cows and goats provided a degree of zooprophylaxis [42, 53]. Moreover, the animals also disturbed local water sources which inhibited mosquito breeding [54]. When Dayaks contracted fever, they applied cold bath rituals and took traditional medicines that had some anti-malarial activity [55–57]. Nevertheless, the estimated malaria-associated death rate did not change after the introduction of quinine [31, 36], suggesting that its distribution did not reach the interior of Kalimantan.

In the pre-Independence era (before 1945), a specific vector control strategy was implemented in Indonesia with beneficial results [58]. This was called ‘species sanitation’ by Swellengrebel [59], a Dutch physician, and consisted of ‘a naturalistic approach of vector control, directed against the main vectors, through modification of the habitat in such a way that the vectors avoid these areas’ [40, 60]. In North Sumatra, for example, drainage of fish ponds, the main breeding site for *An. sundaicus*, reduced local spleen rates from > 90 to 10% [61]. Another example was from West Java, where synchronized rice planting and harvesting attenuated *Anopheles aconitus* breeding in rice paddies and drainage ditches, and decreased malaria transmission to the point where it was no longer a significant health problem in contrast to other parts of the country. Species sanitation was facilitated by documentation of region-specific vector prevalence, starting with Swellengrebel in 1919 [62] and updated by Soesilo in 1936 [40, 63]. In areas of Kalimantan where the main malaria vectors were known (Table 1), malaria outbreaks were successfully managed using this approach.

**Table 1:** Mosquito vectors in Kalimantan from the Dutch era to shortly after independence in 1945. Data modified from [40]

*Anopheline* species	Area	Year of discovery	Habitat
*An. leucosphyrus*	North Kalimantan	1932	Found in brackish water habitats, well-shaded
*An. umbrosus*	Sanggau, Kalimantan	1953	Found in all parts of its range
*An. umbrosus*	Sungai Kakap, Kalimantan	1938	
*An. umbrosus*	Sanggau, Kalimantan	1932	
*An. balabacensis*	Kalimantan (unspecified)	1953	Shy habits and late flight period
*An. flavirostris*	Poelau Laoet, Kalimantan	1938	Typical slow running water breeder
*An. roperi* and *An. letifer*	Sanggau, Kalimantan	1921	
*An. barbirostris*	Kalimantan (unspecified)	1935	Preference for fresh water breeding places mainly fish ponds and rice fields, shade and vegetation loving

After Independence, the newly established Indonesian Ministry of Health recognized the social and economic impact of malaria which was a threat to over a third of the population [64]. New control efforts were implemented based on widespread use of insecticides (including dichloro-diphenyl-trichlorethane (DDT) and dieldrin) and chloroquine. In parts of Indonesia other than Kalimantan such as Java, South Sumatra, Northern Central Sulawesi and Maluku [65–67], DDT spraying was introduced along with mass chloroquine treatment [68]. DDT control of *An. aconitus*, *Anopheles subpictus* and *An. sundaicus* [69] led to a marked reduction in malaria hospitalizations in Java, Sumatra and Sulawesi [70]. Unfortunately, the targeted effect of the species sanitation approach on mosquito vectors contrasted with the potentially devastating impact of DDT spraying on local insect populations. Kalimantan did not have the same degree of wet agriculture as other areas of Indonesia and so was not initially included in the new control programme.

Supported by the WHO and United States Agency for International Development, the Indonesian Government next launched a malaria eradication programme between 1959 and 1968 which was still based on the use of DDT and chloroquine [71]. This strategy became increasingly ineffective because malaria parasites developed resistance to chloroquine monotherapy and mosquitoes became tolerant or resistant to DDT and dieldrin, against a background of financial restraints as well as political unrest in the mid-1960s [40]. The WHO effectively abandoned eradication as a goal in 1969 [72]. From 1969 to 1999, the eradication programme in Indonesia was replaced by a malaria control programme under the Indonesian Directorate General of Communicable Disease Control. Due to population density and economic considerations, the programme focused heavily on Java and Bali. Malaria control in Kalimantan consisted solely of passive case detection at village level Public Health Centres (PHCs) and malariometric surveys in provinces where there was high rates of migration and new economic development projects. Chemoprophylaxis was reserved only for visitors and migrants to Kalimantan, and primaquine was not available to reduce the burden of *P. vivax* infection [7, 11].

The malaria control programme of 1969–1999 reduced the incidence of malaria significantly in western Indonesia but challenges remained in the outer islands including Kalimantan due to poor infrastructure, limited human resources and logistic difficulties in accessing at-risk populations. In 2000, the strategy changed to a Roll Back Malaria campaign with case mapping, identification of foci of transmission, and tailoring of interventions to local needs [2, 7]. A particular aim was to minimize the gap between western and eastern Indonesia and to achieve staged elimination for the whole country [9]. For Kalimantan, elimination by 2020 was planned [8]. In recent years, the numbers of malaria cases in Kalimantan, apart from East Kalimantan, have been generally low [11], but the emergence of *P. knowlesi* in neighbouring Sabah and Sarawak demonstrate a potential new threat to Indonesia’s aspirations for malaria elimination [73].

### Prevalence of malaria in Kalimantan

After the landmark epidemiological studies of Nieuwenhuis in the late 1800s [74], subsequent reports of malaria prevalence in Kalimantan were from West Kalimantan in the 1970s at a time when the logging industry was flourishing [75]. The first formal scientific assessment of malaria in Kalimantan was published at this time as a part of wider parasitological survey of the Indonesian archipelago [76]. Of 5773 samples taken in 15 villages, slide positivity rates (SPRs) were between 4.4% [76] and 5.6% [77]. Most cases were in children and young adults, and the infecting species were *P. vivax* (predominant), *P. falciparum,* or both. In Central Kalimantan in the 1980s, malaria was found in forest workers in two logging companies with SPRs of 7.9% and 14.5% [78]. In East Kalimantan, the SPR in Atap village was 59.7% (*P. falciparum* in all 94 cases including one mixed infection with *P. vivax*) in 2008, and the SPR in Lubakan village at around the same time was 97.3% (by contrast, all but one of 183 cases were *P. vivax*) [79]*.* In South Kalimantan, data from PHCs at Muara Uya and Santuun showed the predominant species to be *P. falciparum* (79% at Muara Uya and 44% at Santuun) followed by *P. vivax* (10% and 23% respectively) with mixed *P. falciparum*/*P. vivax* infections accounting for the remainder [80]. In South Kalimantan, a survey of people living near cattle sheds showed 12% *P. falciparum* and 88% *P. vivax* amongst a total of 235 cases [81]. In four villages in Nunukan, East Borneo, an area bordering Malaysian Borneo, SPRs varied from 0.9 to 5.60%, all *P. falciparum* [82], although mixed infections with *P. vivax* have also been found in this area [79]. A mass blood survey and passive case detection in forested areas of Central and South Kalimantan revealed SPRs between 1.4% and 3.0%, with *Plasmodium malariae* identified in addition to *P. falciparum* and *P. vivax* [83]. In Kotabaru [84] and Tanah Bumbu districts [85], South Kalimantan, active mass blood surveys found SPRs of 23.0% and 1.4%, respectively, with *P. falciparum, P. vivax* and some mixed *P. falciparum/P. vivax* infections.

These data, from a variety of sources published between 1975 and 2005, and utilizing a range of epidemiological survey methods at 17 locations generating 7,367 blood film examinations, show that (i) the SPR is heterogeneous between and within the provinces of Kalimantan, ranging from low level transmission (< 6.0%) in parts of South, East, West and Central Kalimantan to very high (> 50%) in areas of East Kalimantan (there are no published data from North Kalimantan), (ii) although overall *P. falciparum* is the predominant infecting species (5.4% of all blood film examinations [7, 76, 86–88]), *P. vivax* is also regularly encountered (3.4%) alone and in a small minority of mixed infections with *P. falciparum*, and a few (0.3%) *P. malariae* infections have been reported, and (iii) although no cases of *P. knowlesi* have been detected in the publications reviewed (which does not include case reports, as detailed below), it is possible that this parasite has been misdiagnosed by microscopy not only as *P. malariae* since the blood stages of these two species have similar morphology but also as *P. falciparum* or *P. vivax*, as observed previously in Malaysian Borneo [18, 89, 90].

**Fig. 2:**
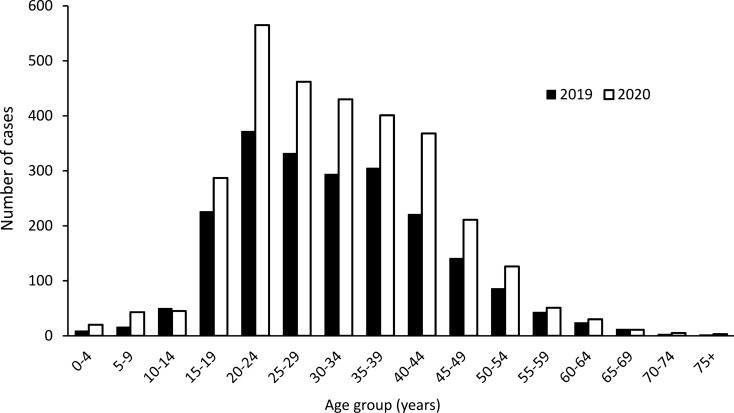
Age distribution of malaria notifications in Kalimantan in 2019 and 2020

To obtain contemporary estimates of malaria prevalence, unpublished data from the Indonesian Health Department were obtained to complement limited published sources. These are summarized in Tables 2 and 3, and in Fig. 2, and confirm that the highest transmission is in East Kalimantan. The case numbers appear stable (allowing for partial data in 2021) over the last 4 years. There is a slight predominance of *P. vivax* over *P. falciparum*, a relatively small number of cases of *P. malariae* (which given the rarity of testing by PCR, as noted as above, may be *P. knowlesi*) and single cases of *Plasmodium ovale* and *P. knowlesi* (the latter presumably having been confirmed by PCR). Most cases are in adult males, consistent with occupational exposure through agriculture and forest-related activities [27, 91].

**Table 2:** Number of malaria cases by province in Kalimantan. Data are from [9]

Province	2018	2019	2020	2021*
West Kalimantan	3	22	64	7
Central Kalimantan	471	202	130	49
South Kalimantan	139	861	535	129
East Kalimantan	2314	2065	2129	949
North Kalimantan	17	56	67	17
Total	2944	3206	2925	1151

^*^Incomplete reporting for this year

**Table 3:** Number of malaria positive cases detected by Public Health Centres. Data are from [11]

Year		2019	2020
Province	West Kalimantan	28	65
	Central Kalimantan		
	South Kalimantan	877	1031
	East Kalimantan	2138	2395
	North Kalimantan	65	68
Species	*P. falciparum*	1066	1188
	*P. vivax*	1535	1303
	*P. falciparum* and *P.vivax*	440	436
	*P. malariae*	44	46
	*P. ovale*	1	0
	*P. knowlesi*	1	0
Diagnostic method	Rapid Diagnostic Test	970	899
	Microscopy	2116	2155
	PCR confirmation in central laboratory	1	0
Sex	Male	2817	2862
	Female	291	199

### Mosquito vectors in Kalimantan

The first published literature on mosquito vectors in Kalimantan appeared during the implementation of the species sanitation control strategy in the early twentieth century. The Dutch physician Swellengrebel pioneered the identification of Indonesian anopheline mosquitoes in 1919 [62] and described *Anopheles roperi* and *Anopheles letifer* in Sanggau, West Kalimantan in 1921 and 1932 [40]. The recognition of *Anopheles barbirostris* as an important vector species in Sumatra, Java and Kalimantan, and the introduction of water vegetation-eating fish *Puntius javanicus* as an effective measure against water surface plant-loving mosquitoes by Walch followed in 1935 [58]. Indonesian physician Soesilo compiled an updated list of vectors in 1936 [63] which has proved a valuable tool in reducing the burden of malaria in endemic areas of Indonesia including Kalimantan [58] (Table 1).

A more recent 1987 study of *Anopheles* mosquitoes collected in South Kalimantan using human bait determined that *Anopheles leucosphyrus* and *Anopheles balabacensis* (97.7% of the 217 female specimens) were malaria vectors, with sporozoite infection rates of 1.0% and 1.3%, respectively [92]. A subsequent study used malaria case mapping and examined mosquito vector species and habitats in Sebatik Island in East Kalimantan in 2009. *An. balabacensis, An. sundaicus* and *Anopheles maculatus* were identified as vectors, while *An. balabacensis* and *An. maculatus* were found to be resistant to the insecticides permethrin and lambdacyhalothrin, and tolerant to malathion [82]. In 2012, in a study of the efficacy of vector control strategies in Nunukan, East Kalimantan [93], the habitat characteristics of *Anopheles spp.* larvae indicated that potential breeding places were dominated by fish ponds with stagnant water and muddy substrate, located around settlements and surrounded by grasses, shrubs and trees [94]. Another vector identification study in Sebatik Island, North Kalimantan, revealed that *Anopheles vagus, An. sundaicus* and *An. subpictus* were the most abundant species caught and that both *Anopheles peditaeniatus* and *An. sundaicus* were *P. falciparum* vectors [95]. A mosquito survey in Kotabaru using nested PCR assays found that *P. vivax* was present in *Anopheles vagus, An. peditaeniatus* and *Anopheles tesselatus,* indicating that they were new malaria vectors in South Kalimantan [96].

**Fig. 3:**
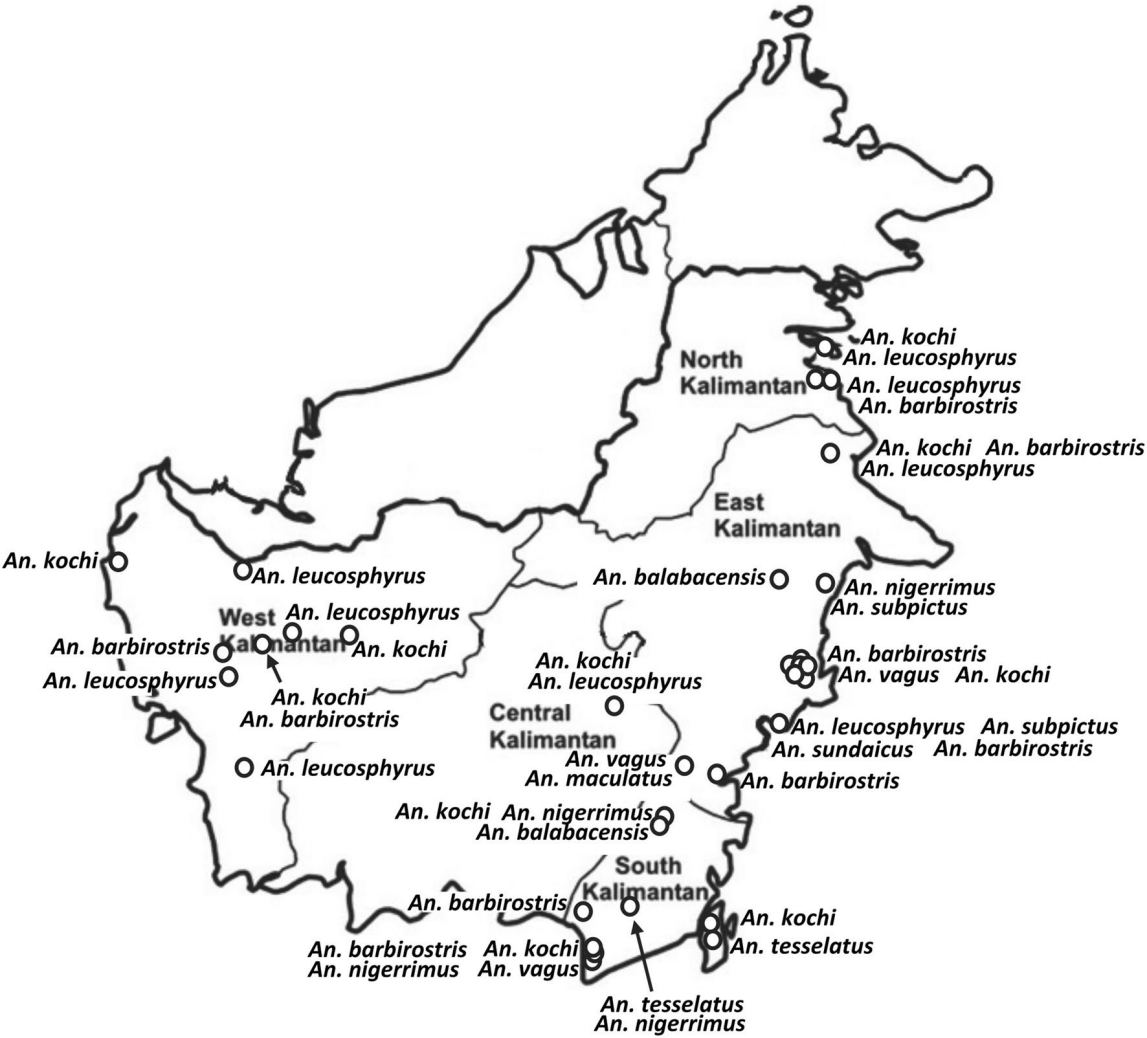
Mapping of *Anopheles* spp*.* distribution in Indonesian Kalimantan (adapted from Elyazar et al. [69])

Data on malaria mosquito vectors and insecticide resistance of *Anopheles spp.* in Kalimantan from a few published sources but mainly from unpublished Indonesian Health Department (*Balitbangkes*) and Global Fund reports are summarized in Tables 4, 5 and Fig. 3. These indicate that the vectors of *P. knowlesi* and other simian malaria parasites in Malaysian Borneo such as *An. balabacensis*, *Anopheles latens* and *Anopheles umbrosus* are present in Kalimantan [97, 98].

**Table 4:** *Anopheles spp* confirmed as malaria vectors in Kalimantan. Adapted from [69]

No.	Collected	Tested	Province	District	Vector species
1	2016	2016	South Kalimantan	Barito Kuala	*An. subpictus*
2	2016	2016	South Kalimantan	Barito Kuala	*An. indefinitus*
3	2017	2017	Central Kalimantan	Pulang Pisau	*An. barbumbrosus*
4	2017	2017	Central Kalimantan	Pulang Pisau	*An. barbirostris*
5	2017	2017	Central Kalimantan	Gunung Mas	*An. letifer*
6	2017	2017	Central Kalimantan	Gunung Mas	*An. barbumbrosus*
7	2017	2017	Central Kalimantan	Gunung Mas	*An. kochi*
8	2017	2017	Central Kalimantan	Gunung Mas	*An. nigerrimus*
9	2017	2017	Central Kalimantan	Gunung Mas	*An. latens*
10	2017	2017	Central Kalimantan	Murung Raya	*An. umbrosus*
11	2017	2017	Central Kalimantan	Murung Raya	*An. letifer*
12	2017	2017	Central Kalimantan	Murung Raya	*An. vagus*
13	2017	2017	Central Kalimantan	Murung Raya	*An. barbirostris*
14	2010	2016	North Kalimantan	Nunukan	*An. peditaeniatus*
15	2010	2016	North Kalimantan	Nunukan	*An. sundaicus*

**Table 5:** Insecticide resistance of anopheline species in Kalimantan. Data are courtesy of Elyazar IE [69]

Insecticide	Dosage	Location	Year tested	Species tested
Malathion	0.80%	West Banjarmasin, South Kalimantan	2010	*Ae. aegypti*
		Central Banjarmasin, South Kalimantan	2010	*Ae. aegypti*
		South Banjarmasin, South Kalimantan	2010	*Ae. aegypti*
		North Banjarmasin, South Kalimantan	2010	*Ae. aegypti*
		West Banjarmasin, South Kalimantan	2010	*Ae. aegypti*
		West Pontianak, West Kalimantan	2002	*Ae. aegypti*
		West Kalimantan	2005	*Ae. aegypti*
	5%	West Kalimantan	2004	*Ae. aegypti*
	0.50%	West Kalimantan	2004	*Ae. aegypti*
Lambda Cyhalothrin	0.05%	East Kalimantan	2004	*An. vagus*
		East Kalimantan	2004	*An. koci*
		East Kalimantan	2004	*An. tesselatus*
		East Kalimantan	2004	*An. pediteniatus*
		East Kalimantan		*An. kochi*
		East Kalimantan		*An. peditaeniatus*
		East Kalimantan		*An. tesselatus*
		East Kalimantan		*An. vagus*
Etofenprox	0.50%	West Kalimantan	2005	*An. nigerimus*
		West Kalimantan		*An. nigerrimus*
		Penajam Paser Utara, East Kalimantan		*An. hyrcanus*
Deltamethrin	0.05%	West Kalimantan	2005	*An. nigerimus*
		West Kalimantan		*An. nigerrimus*
		Angkinang, Hulu Sungai Selatan, South Kalimantan		*An. nigerrimus*
		Dedai, Sintang, West Kalimantan		*no data given*
		Muara Aya, Tabalong, South Kalimantan		*An. vagus*
		Muara Aya, Tabalong, South Kalimantan		*An. barbirostris*
		Timpah, Kapuas, Central Kalimantan		*An. letifer*
		Sebatik, Nunukan, East Kalimantan		*An. sundaicus*
Permethrin	0.75%	Dedai, Sintang, West Kalimantan		*An. vagus*
		Muara Aya, Tabalong, South Kalimantan		*An. vagus*
		Sebatik, Nunukan, East Kalimantan		*An. sundaicus*
Bendiocarb	0.10%	Dedai, Sintang, West Kalimantan		*An. peditaeniatus*
		Muara Aya, Tabalong, South Kalimantan		*An. vagus*
		Muara Aya, Tabalong, South Kalimantan		*An. kochi*
		Muara Aya, Tabalong, South Kalimantan		*An. barbirostris*
		Muara Aya, Tabalong, South Kalimantan		*An. vagus*
		Muara Aya, Tabalong, South Kalimantan		*An. kochi*
		Muara Aya, Tabalong, South Kalimantan		*An. barbirostris*
		Sebatik, Nunukan, East Kalimantan		*An. sundaicus*
Fenitrotion	1%	Muara Aya Tabalong, South Kalimantan		*An. vagus*
		Dedai, Sintang, West Kalimantan		*An. peditaeniatus*
		Timpah, Kapuas, Central Kalimantan		*An. letifer*
		Sebatik, Nunukan, East Kalimantan		*An. sundaicus*
DDT	4%	Sebatik, Nunukan, East Kalimantan		*An. sundaicus*

### Non-human primate malaria

The first non-human primate malaria species in Borneo were identified by Halberstaedter and Prowazek in 1907 [99]. The blood of *Macaca irus* and *Macaca nemestrina* imported from Indonesian Borneo harboured a new species of parasite *Plasmodium inui* (after the old generic name of the primate host *Inuus*)*.* This parasite was also found in cynomolgus monkeys (*Macaca fascicularis)*. *Plasmodium pitheci* was discovered in the same year in the blood of an orang-utan (*Pongo pygmaeus*) and *Plasmodium hylobati* was detected in gibbon blood from a mature male *Hylobates moloch*, both from Borneo [100]. These two latter parasites are known to be host specific, but human infection with *Plasmodium cynomolgi* has been reported from Malaysian Borneo [101]. *Plasmodium inui* can also infect humans [102] and its sporozoites have been found in *Anopheles* mosquitoes in Sabah, Malaysian Borneo [103].

Three other species of malaria that can be harboured by macaques in Malaysian Borneo are *Plasmodium fieldi* [104], *Plasmodium coatneyi* [105] and *Plasmodium simiovale* [106]. *Plasmodium fieldi* and *P. coatneyi* have been found in *An. balabacensis* Baisa in Sabah [103], while *P. simiovale* was detected in long-tailed macaques in Sarawak [107]. Both *P. fieldi* and *P. coatneyi* human infections have been found outside Borneo in Peninsular Malaysia, and human *P. simiovale* infections have been detected in Peninsular Malaysia and Sarawak, Malaysian Borneo [102].

The predominant simian malaria species that infects humans in Malaysian Borneo is *P. knowlesi* [108]. Malaysia is the nation with the highest reported prevalence of *P. knowlesi* in South-east Asia [109, 110] and, in Sabah and Sarawak, nearly 80% of malaria cases are caused by *P. knowlesi* [111] which is also the predominant species responsible for malaria hospitalizations [112, 113]. Given that it shares borders with Sabah and Sarawak, it is important to determine whether knowlesi malaria is also found in Kalimantan. Reports of *P. knowlesi* in humans from Kalimantan are scant, in part because the species is not included as part of routine malaria diagnostic screening in PHCs. The diagnosis of *P. knowlesi* has not normally been considered, except in a few suspected cases where identification was performed in a laboratory outside Kalimantan. In the first such report, 4 out of 22 human blood samples from Kalimantan patients with malaria were identified by molecular methods as containing *P. knowlesi* (two of them mixed infections with *P. falciparum* or *P. vivax*) by a laboratory in Germany in 2009 [114]. A second report was of an Australian working in South Kalimantan with the diagnosis made by PCR in a Sydney laboratory [115]. A third report involved PCR analysis of 287 filter paper dried blood spots from malaria patients in Central and South Kalimantan, three of which (1.1%) were positive for *P. knowlesi* [116]. A fourth report in 2016 of *P. knowlesi* was the case of a 60-year-old man working at a coal mining site in Central Kalimantan [117].

**Fig. 4:**
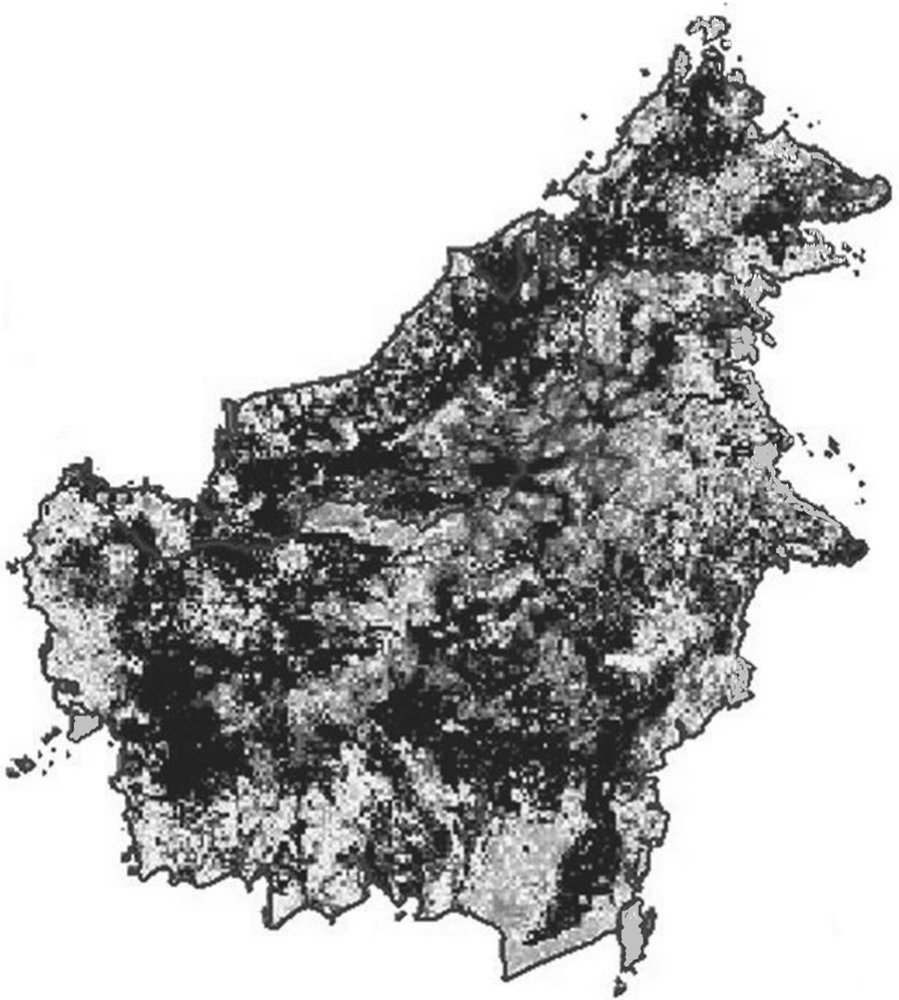
Map of *Plasmodium knowlesi* malaria risk on the island of Borneo as adapted from Shearer et al. [73]. The darker the shaded area, the higher the risk

The limited number of *P. knowlesi* cases both in the literature and recent Indonesian Health Department data contrast with the relatively large numbers seen in Sarawak [15–18] and Sabah [19–21]. Long-tailed and pig-tailed macaques are present in Kalimantan [118] and so are the vectors capable of transmitting knowlesi malaria [69, 118]. Kalimantan is within the geographic range of the species required for *P. knowlesi* zoonotic transmission and therefore estimated as a high-risk area in *P. knowlesi* risk-prediction mapping, indeed as high as in Malaysian Borneo (Fig. 4). This may mean that the distribution of vectors, environmental conditions facilitating transmission and/or methods of accurate detection of the parasite are different in Kalimantan compared with Malaysian Borneo. Unfortunately, data relating to the prevalence of malaria parasites in macaques in Kalimantan are lacking. There are non-human primates in Kalimantan other than *Macaca*, *Pongo* and *Hylobates*, from genus *Nycticebus, Cephalopachus, Presbytis, Trachypithecus, Nasalis* [119], but it is not known whether they can harbour malaria.

### *Plasmodium* parasite genetic diversity

A study in Ketapang district of West Kalimantan found that *P. falciparum* genetic diversity was low and its linkage patterns were consistent with unstable transmission and therefore amenable to targeted intervention; *P. vivax* diversity was higher and transmission seemed to be more stable [120]. Two studies of *P. vivax* genetic diversity in South and Central Kalimantan demonstrated that the Kalimantan parasite had distinct haplotypes due to mutation, recombination, and positive selection [121] and that there was differences in merozoite surface protein-1 gene (*Pvmsp-1*) between migrant and indigenous Dayak people [122].

### Anti-malarial therapy and drug resistance

Choice of anti-malarial therapy in Kalimantan is as contained in national guidelines [123] which have been developed from studies in the eastern part of Indonesia [124–126]. First-line treatment for any malaria species is dihydroartemisinin-piperaquine (DHP) and primaquine. For complicated severe malaria, parenteral artesunate or quinine are recommended [123]. Before the WHO recommendation of artemisinin-based combination therapy (ACT) was widely implemented, chloroquine was used as monotherapy in Kalimantan. Some studies in Kalimantan between 1975 and 1991 found chloroquine-resistant malaria [88, 127–130] and many patients experienced relapse or recrudescence [88, 127]. In 1991, the first line treatment for malaria in Kalimantan was still chloroquine followed by primaquine, with sulfadoxine-pyrimethamine (SP) second line and quinine third line [7].

In 2004, artesunate plus amodiaquine (AS-AQ) was recommended as the first line therapy for *P. falciparum* infection only, followed by quinine and doxycycline as second line, and by quinine and tetracycline as the third line [7, 123]. Intramuscular artemether was also recommended for severe complicated falciparum malaria. Other malaria species were still to be treated with chloroquine and primaquine. These changing and complex treatment recommendations may have caused confusion amongst health professionals. In 2007, the government recommendation was that all malaria cases should be treated with an ACT, either AS-AQ or DHP, apart from *P. malariae* and *P. ovale* infections, and chemoprophylaxis, where chloroquine and doxycycline were used, respectively [2, 123]. The current recommendation from 2017 is, however, to use ACT for all malaria cases as a result of several drug safety and efficacy studies in Papua, Indonesia [124]. Chloroquine is no longer available as an anti-malarial. After the introduction of ACT, several studies took place in Kalimantan to assess the safety and efficacies of DHP and ASAQ in *P. falciparum, P. vivax* or both *P. falciparum* and *P. vivax* in malaria patients in Central and West Kalimantan with positive results [131–133].

*Plasmodium falciparum* resistance to chloroquine was identified in vitro in the early 90s in Nunukan and in vivo in Banjarmasin, both in East Kalimantan [87, 134]. *Plasmodium vivax* resistance to chloroquine has since been identified in the Ketapang district of West Kalimantan [86]. The chloroquine-resistant East Kalimantan *P. falciparum* strains were sensitive to other anti-malarial drugs such as halofantrine, mefloquine and artesunate [135–138]. A summary of studies of parasite resistance to standard anti-malarial medications before ACT was introduced In Indonesia is shown in Table 6. In 2012, molecular studies were performed in Kalimantan to determine the mutations in *P. falciparum* and *P. vivax* that cause resistance [139, 140]. Another study analysed mutations in *P. falciparum* dihydrofolate reductase and dihydropteroate synthase in South Kalimantan samples from pregnant women [141] and in adults [142]. In these studies, there was evidence of previously reported mutations in *P. falciparum* and *P. vivax* associated with resistance to chloroquine and SP.

**Table 6:** Drug resistance in Kalimantan

	Anti-malarial drugs	Year of sample	In vivo studies			In vitro studies		
			No. sites	No. examined	No. resistant (%)	No. sites	No. examined	No. resistant (%)
*P. falciparum*	Chloroquine	1973–2000	20	157	62 (39%)	11	319	182 (57%)
	Sulfadoxine-pyrimethamine	1987–1991	–	–	–	4	136	109 (80%)
	Quinine	1990–1995	–	–	–	3	142	5 (4%)
*P. vivax*	Quinine	1998	1	27	12 (44%)	–	–	–

Table is modified from Elyasar et al. [7]

A study of traditional falciparum malaria remedies performed in Kenyah indigenous people of the Apo Kayan region in East and North Kalimantan showed evidence of anti-malarial activity [55]. More recently, in 2021, two traditional treatments for *P. falciparum, Cratoxylum sumatranum* and *Garcinia parvifolia*, were analysed in Balikpapan Botanical Garden in East Kalimantan [143, 144] and the extracts also showed anti-malarial activity. The potential role of such therapies in the treatment of malaria needs further validation.

### Current malaria control efforts in Kalimantan

Since the Roll Back Malaria programme was implemented in 2000, Kalimantan has been targeted for elimination by 2020 (Stage 3) [2, 9] with an increased emphasis on control measures such as enhanced case detection and improved diagnostic accuracy [145]. Even though elimination has not yet been achieved, there has been a substantial reduction in the burden of malaria in Kalimantan during the last two decades so that there are generally low rates of local transmission [9]. The API for South, West, North and Central Provinces was 0.13 in 2020 while it was 1.23 in East Kalimantan [11]. Some studies in Kalimantan assessing diagnostic accuracy have suggested the possibility of malaria being underdiagnosed. A qualitative study involving 10 PHCs in Sambas, West Kalimantan, found that inadequate laboratory facilities and human resources are associated with low compliance with malaria service delivery including blood film examination in suspected cases [146]. Nevertheless, the reliability of diagnosis by microscopy seemed high in differentiating positive from negative blood films, but less in determining *Plasmodium* species [147]. Quantifying parasite densities can, if available, provide useful prognostic information in *P. falciparum* (if not for *P. vivax*) infections, with a study from Kalimantan showing that this is particularly useful in children aged under 15 years [148].

Regarding malaria control efforts such as the usage of long-lasting insecticidal nets (LLINs), a study in Sungai Nyamuk village, Nunukan district, East Kalimantan, analysed the effectiveness of LLINs against *Anopheles* spp. [149]. The result was that the most effective LLIN was one that had been used for up to 6 months with those still in use at 12–24 months were much less effective, although it needs to be acknowledged that LLINs will have limited impact where local vectors feed in the early evening and outdoors. Identifying foci of malaria transmission is important in targeting malaria control strategies. In a spatial analysis study of recorded malaria cases in high-risk villages in the northern part of Kotabaru, South Kalimantan, two persistent and four re-emerging high-risk clusters were identified, confirming its potential role in informing the efficient deployment of limited malaria control resources [150].

## Conclusions

Kalimantan is a large forested landmass with a substantial number of inhabitants living in heterogeneous environmental conditions ranging from densely populated coastal towns to sparsely populated logging regions, palm oil plantations and mining areas. There are water bodies where mosquitoes breed and dense forested areas where different species of primates thrive, including macaques (the hosts for *P. knowlesi*), Most people in Kalimantan are at risk of malaria despite the fact that parasite species distributions and prevalence rates vary substantially across the provinces in line with differences in population density and mobility, and the presence of mosquito vectors [4, 5, 69]. As a result, malaria control efforts need to be tailored to the local situation in order to be successful. Although the malaria situation has improved little over the last few years, there has been progress in Kalimantan since Roll Back Malaria was instituted in 2000.

The large number of human *P. knowlesi* infections seen in Malaysian Borneo has no parallel in Kalimantan. This is despite the presence of anopheline vectors known to transmit *P. knowlesi* [97] and long-tailed macaques [151]. Interestingly, *P. knowlesi* has not been detected in wild macaques in Indonesia [152] even though some human cases have been reported. This may reflect limited screening studies to date or sampling in areas of Indonesia with relatively low primate transmission. Indeed, the recent reduction in human malaria cases in Indonesia has resulted in more cases of simian malaria, especially due to *P. knowlesi*, in Sumatra [153]. It is possible that better diagnostic testing through more widely available PCR (including correctly identifying *P. malariae* as *P. knowlesi*) could unmask a more significant number of knowlesi malaria cases in Kalimantan. The role of deforestation and climate change may increase human contact with vectors carrying *P. knowlesi* in areas of Kalimantan in future. Given the potentially lethal nature of knowlesi malaria [154], efforts to ensure that it is diagnosed promptly and accurately should be a priority.

## Supplementary Information

**Additional file 1: Table S1.**Description of study sites and number of samples collected at each.** Figure S1.** Neighbour-Joining phylogenetic trees of Plasmodium-genus positive samples isolated from Kapuas Hulu, West Kalimantan, Indonesia based on the small subunit ribosomal RNA genes. Nucleotide sequences from the isolates are in bold, and were generated using (A) primers rPLU3 and rPLU4 with ~240 bp in length, and (B) primers Plasmo1 and Plasmo2 with ~252 bp in length. The bootstrap values at nodes were generated by 1,000 replicates, and only values above 70% are shown. In this result, the identity of the infecting Plasmodium species (samples in bold) could not be inferred through phylogenetic analyses since the DNA sequences generated were relatively short, resulting in phylogenetic trees with low bootstrap values. Samples KI 353 and KI 353 are from Participant no. 7, KI 809 is from Participant no. 8, KI 59 is from Participant no. 9, KI 334A and KI 334B are from Participant no. 10, KI 175A, KI 175B and KI 175 are from Participant no. 11, KI 978 is from Participant no. 15, and KI 676 is from Participant no. 16.

## Data Availability

The datasets used and/or analysed during the current study are available from the corresponding author on reasonable request.
